# Challenges in diagnosis and management of aortobronchial fistula: a case report

**DOI:** 10.1093/ehjcr/ytae437

**Published:** 2024-09-01

**Authors:** Mohamed Hamza Abaydi, Safae Dhimene, Amine Ech-chenbouli, Badre El Boussaadani, Zainab Raissuni

**Affiliations:** Cardiology Department, Mohamed VI University Hospital of Tangier; Abdelmalek Essaadi University Faculty of Medicine and Pharmacy, Street of Rabat, Km 17, BP 398, 90100 Tangier, Morocco; Cardiology Department, Mohamed VI University Hospital of Tangier; Abdelmalek Essaadi University Faculty of Medicine and Pharmacy, Street of Rabat, Km 17, BP 398, 90100 Tangier, Morocco; Cardiology Department, Mohamed VI University Hospital of Tangier; Abdelmalek Essaadi University Faculty of Medicine and Pharmacy, Street of Rabat, Km 17, BP 398, 90100 Tangier, Morocco; Cardiology Department, Mohamed VI University Hospital of Tangier; Abdelmalek Essaadi University Faculty of Medicine and Pharmacy, Street of Rabat, Km 17, BP 398, 90100 Tangier, Morocco; Cardiology Department, Mohamed VI University Hospital of Tangier; Abdelmalek Essaadi University Faculty of Medicine and Pharmacy, Street of Rabat, Km 17, BP 398, 90100 Tangier, Morocco

**Keywords:** Thoracic endovascular aortic repair (TEVAR), Aortobronchial fistulas (ABFs), Haemoptysis, Anticoagulation, Tranexamic acid, Heart failure, Case report

## Abstract

**Background:**

Aortobronchial fistulas (ABFs) are rare but potentially life-threatening conditions, often presenting with haemoptysis. They can develop following various thoracic aortic conditions or procedures.

**Case Presentation:**

A 70-year-old patient with a history of descending aorta replacement and ischaemic stroke presented with chest pain and upper gastrointestinal bleeding. Imaging revealed a fistula between the aortic prosthesis and the lung, along with other cardiovascular abnormalities. Despite the indication for anticoagulant therapy, tranexamic acid was initiated due to bleeding risk. The patient showed clinical improvement with tranexamic acid treatment but experienced recurrence of bleeding after discontinuation. Endovascular treatment for the contained rupture at the proximal stent anastomosis was indicated.

**Discussion:**

Haemoptysis is the primary symptom of ABFs, often recurring until the fistula enlarges. Postoperative aortic fistulas into the airways are uncommon and can occur years after surgery. Thoracic endovascular aortic repair has become the primary treatment for high-risk patients with thoracic aortic disease. Various diagnostic modalities can visualize a fistula tract, but practical visualization is rare. Untreated ABFs invariably lead to death.

**Conclusion:**

This case highlights the challenges in diagnosing and managing ABFs, emphasizing the need for a multidisciplinary approach and regular follow-up. Patient education and prompt reporting of symptoms are essential. Early intervention upon suspicion of recurrence is crucial for optimizing patient outcomes.

**Learning Points**

- Aortobronchial fistulas (ABFs) are rare but potentially life-threatening complications that can occur following thoracic aortic surgery, such as thoracic endovascular aortic repair (TEVAR).
- Management of ABFs requires balancing the risks of bleeding and the need for anticoagulation in patients with concomitant cardiac issues. Surgical repair or endovascular stent-graft placement is necessary treatments depending on the size and location of the fistula.

## Introduction

Aortobronchial fistulas (ABFs) are rare and late complications of cardiac surgery that can be fatal if left untreated. They most often occur after procedures involving the descending thoracic aorta. Haemoptysis, which can be massive or intermittent, is the primary symptom. The interval between the initial surgery and the onset of haemoptysis ranges from 3 weeks to 25 years. Diagnostic examinations are often unable to directly visualize a fistula. Indication for surgical or endovascular repair typically relies on clinical suspicion and non-specific diagnostic features.^1,2^

This case of a 70-year-old patient with a history of descending aorta replacement underscores several critical aspects of healthcare delivery. It highlights the diagnostic challenges and the importance of multimodal imaging in identifying ABFs. Additionally, the management complexities, particularly balancing anticoagulation needs with bleeding risks, are evident. This case also emphasizes the necessity of a multidisciplinary approach and long-term follow-up to optimize patient outcomes and prevent recurrence.

## Summary figure

**Table:** 

Date	Event
1996	TEVAR surgery: descending aorta replacement following aortic dissection.
**June 2023**	Ischaemic stroke: 2 months prior to current admission.
**August 2023**	**1 week before admission**: chest pain and 500 mL of upper gastrointestinal bleeding. Oesophagogastroduodenal endoscopy returned normal. **Day 0**: presentation to the emergency room with chest pain and a second episode of upper gastrointestinal bleeding. Admission to the cardiac intensive care unit. Diagnosis of haemoptysis made instead of haematemesis. Treatment with tranexamic acid. **Day 1**: thoracic computed tomography (CT) angiography performed with suspicion of aortobronchial fistula, confirmed on second reading. **Day 7**: discharged on heart failure treatment and tranexamic acid therapy following clinical improvement and no recurrence of bleeding.
**September 2023**	Cardiac MRI revealed ischaemic heart disease with a left ventricular ejection fraction (LVEF) of 38% and a small thrombus lining the apex. Bronchoscopy showed a diffuse inflammatory appearance.
**November 2023**	Endovascular treatment for the contained rupture at the proximal stent anastomosis was considered in multidisciplinary counselling, involving the surgeon who initially operated on him.
**February 2024**	Decision to discontinue tranexamic acid due to stable condition.
**March 2024**	Recurrence of bleeding, and tranexamic acid was reintroduced.
**May 2024**	Patient awaiting preparation of the most appropriate endoprosthesis.

## Case presentation

A 70-year-old patient with a history of descending aorta replacement and ischaemic stroke presented to the emergency room with chest pain and 500 mL of haematemesis. He had undergone descending aorta replacement for an aortic dissection complicated by a thrombotic aneurysm 27 years prior and had experienced an ischaemic stroke 2 months before this presentation. Following the stroke, the patient was on antiplatelet therapy (Kardegic) and a statin.

One week before hospitalization, the patient underwent an oesophagogastroduodenal endoscopy due to a prior episode of haemorrhage, which did not reveal any source of bleeding or lesions.

Physical examination revealed atypical chest pain, Stage II dyspnoea according to the MRC (Medical Research Council) dyspnoea scale, and Broca’s aphasia. The patient was haemodynamically stable with a blood pressure of 110/70 mmHg and a heart rate of 76 b.p.m. Respiratory examination showed an SpO_2_ of 96% on room air. Neurologically, the patient was stable. He was admitted to the cardiac intensive care unit for close monitoring. The patient subsequently experienced a new episode of bleeding, which was identified as haemoptysis by a physician after careful examination.

Laboratory findings indicated normochromic normocytic anaemia, with a haemoglobin level of 9.8 g/dL (normal range, >12 g/dL). The electrocardiogram revealed a newly identified counterclockwise flutter, occurring with a ventricular rate of 76 b.p.m. Transthoracic ultrasound showed apical akinesia with a left ventricular ejection fraction (LVEF) of 40% and left atrial dilatation. However, femoral coronary angiography could not be performed due to the blockage of the guide at the level of the thoracic aorta.

**Figure 1:**
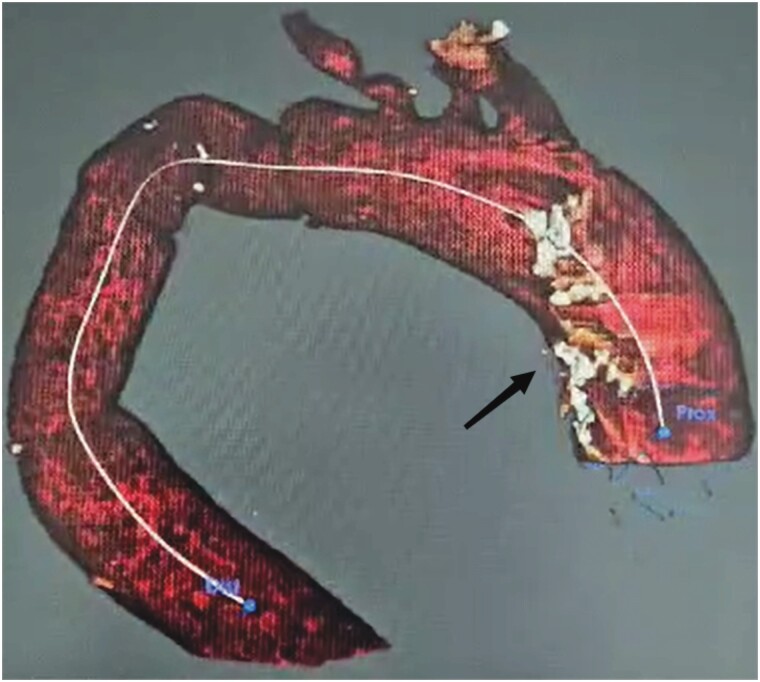
CT angiography with 3D reconstruction of the aorta showing a contained rupture at the proximal anastomosis of the graft.

**Figure 2:**
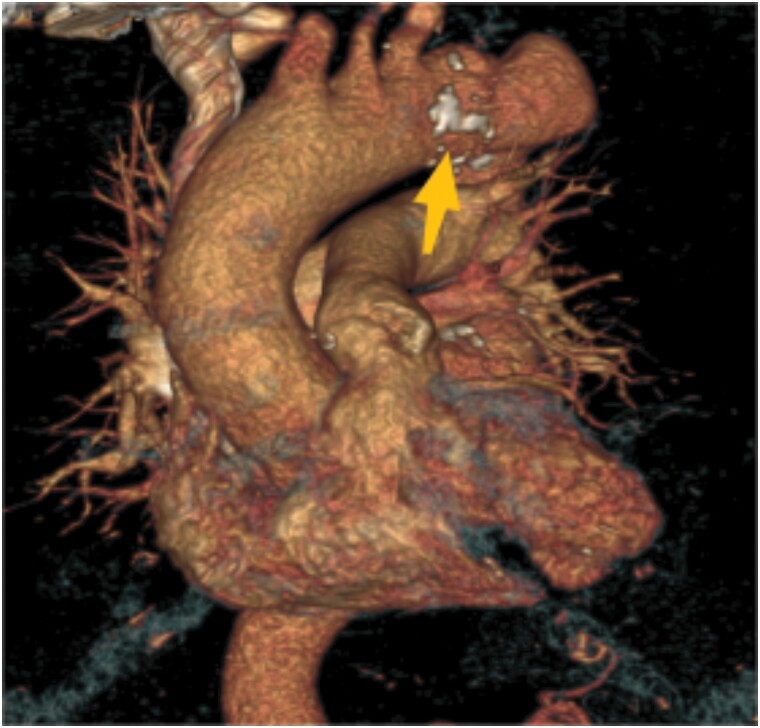
CT angiography with 3D reconstruction of the aorta showing a contained rupture at the proximal anastomosis of the graft.

**Figure 3:**
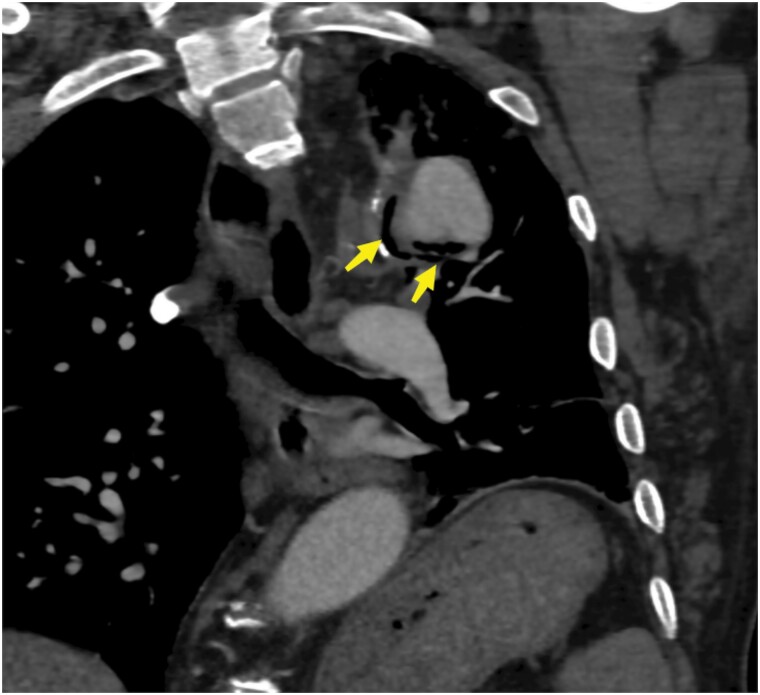
CT angiography in the mediastinal window shows a prosthetic fistula involving the lung.

**Figure 4:**
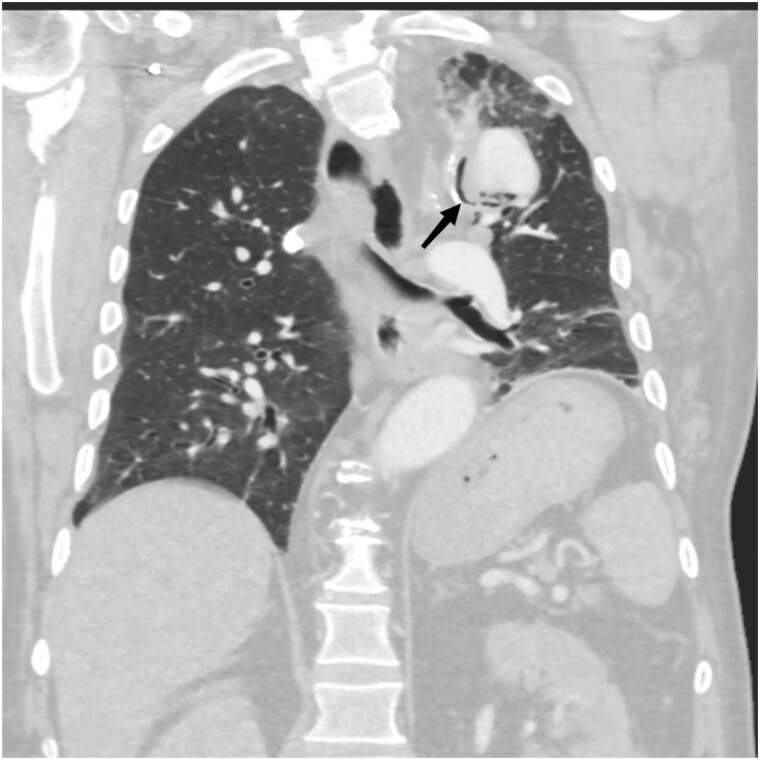
CT angiography in the parenchymal window shows a prosthetic fistula involving the lung.

Thoracic computed tomographic (CT) angiography identified a fistula between the prosthesis and the lung, accompanied by the presence of air and a suspected blood flow during the haemoptysis episode (Figures 1–4). Subsequent bronchoscopy showed a distorted appearance of the entire bronchial tree, with thickened walls and orifices narrowed by extrinsic compression, alongside a diffuse inflammatory appearance.

Cardiac MRI further revealed ischaemic heart disease with segmental kinetic disturbances in the anterior territory, deemed non-viable, an LVEF of 38%, and a small thrombus lining the apex.

Given the patient’s newly identified counterclockwise flutter and the presence of a small thrombus lining the apex, anticoagulant therapy was clearly indicated. However, due to the risk of bleeding, particularly with the episode of haemoptysis, the decision was made to initiate tranexamic acid therapy at a dosage of 500 mg, three times daily. This approach was chosen to mitigate bleeding risk while balancing the need for anticoagulation. The heart failure treatment administered included beta-blockers, spironolactone, angiotensin-converting enzyme (ACE) inhibitors, and SGLT2 inhibitors. Endovascular treatment for the contained rupture at the proximal stent anastomosis was considered during multidisciplinary counselling but was deemed non-urgent due to the patient’s clinical, haemodynamic, and haematologic improvement.

## Follow-up

The patient was treated with tranexamic acid for 5 months while awaiting the preparation of the most suitable endoprosthesis. During this time, significant improvements were observed in both clinical evaluations and echocardiographic assessments. However, 1 month after discontinuing tranexamic acid, the patient experienced streaks of blood in the sputum, indicating a recurrence of bleeding. Tranexamic acid was reintroduced, leading to clinical improvement and the resolution of bleeding episodes.

## Discussion

Haemoptysis is often the initial and sometimes the sole symptom of ABFs. The severity of haemoptysis can range from massive to intermittent, depending on the size of the fistula. Typically, the fistula is small and may be occluded by clots, remaining closed for weeks or months. Once the clots dissolve or dislodge, the fistula reopens, leading to recurrent bleeding. Over time, this process can escalate, causing more significant bleeding as the fistula enlarges. Other symptoms, such as dyspnoea, cough, and chest or back pain, are less common.^1^

Postoperative aortic fistulas, including aortobronchial and aortopulmonary fistulas, are uncommon and typically present as delayed complications after cardiothoracic surgery. According to a comprehensive review by Piceche *et al*., these fistulas are more prevalent following procedures for descending thoracic aortic aneurysms (dTAA) compared to other cardiac and thoracic surgeries. The review noted an interval of 1.5 to 23 years post-dTAA before symptoms appeared. Notably, only one patient in their review had undergone endovascular stent-graft placement.^1^

Thoracic endovascular aortic repair (TEVAR), introduced in the early 1990s, has become the primary treatment for high-risk patients with thoracic aortic disease.^3^

Chiesa *et al*. examined the incidence of aorto-oesophageal (AEF) and aortobronchial (ABF) fistulas following TEVAR. Of 1113 patients who underwent TEVAR from 1998 to 2008, 19 (1.7%) developed AEF or ABF. Among these, 13 (68%) involved the oesophagus, one (5%) affected the left bronchial tree, and five (26%) showed concurrent broncho-oesophageal involvement.^2^

Similarly, Czerny *et al*. analysed data from the European Registry of Endovascular Aortic Repair Complications, which included 4680 TEVAR patients across 14 European centres. They reported a 0.56% incidence of aortobronchial or aortopulmonary fistula following TEVAR. The primary causes of fistula formation included external compression of the bronchial tree, endoleak formation, and additional aortic ischaemia.^4^

Diagnostic modalities such as CT angiography, bronchoscopy, and aortography can be used to visualize the fistula tract. However, Piceche *et al*. found that direct visualization of the fistula was achieved only in rare cases.^1^

Regarding treatment, Piceche *et al*. found that untreated ABFs invariably result in death. All eight untreated patients in their study died from massive haemoptysis.^1^ Additionally, the patient mentioned in Chiesa *et al.*’s review also died when left untreated.^2^

## Conclusion

This case highlights the diagnostic and management challenges of ABFs, emphasizing the need for a multidisciplinary approach. Regular follow-up with appropriate imaging is crucial to detect early recurrence. Patient education on symptom recognition and prompt reporting is essential. Multidisciplinary communication and early intervention if recurrence is suspected are paramount. Comprehensive management strategies are necessary to optimize patient outcomes.

## Data Availability

The data underlying this article will be shared on reasonable request to the corresponding author.

## Lead author biography

Dr Mohamed Hamza Abaydi, originally from Fes, is a resident in the cardiology department at CHU-Med VI in Tangier. He has a strong interest in interventional cardiology and is eager to make a significant contribution to the medical community. Dr Abaydi remains committed to continuing education and research.
